# Altered Venous Blood Nitric Oxide Levels at Depth and Related Bubble Formation During Scuba Diving

**DOI:** 10.3389/fphys.2019.00057

**Published:** 2019-02-21

**Authors:** Danilo Cialoni, Andrea Brizzolari, Michele Samaja, Massimo Pieri, Alessandro Marroni

**Affiliations:** ^1^DAN Europe Research Division, Roseto degli Abruzzi, Italy; ^2^Apnea Academy Research, Padua, Italy; ^3^Department of Health Sciences, University of Milan, Milan, Italy

**Keywords:** nitric oxide, breath-hold diving, diving, free radicals, oxidative stress

## Abstract

**Introduction:** Nitric oxide (NO) plays an important role in the physiology and pathophysiology of diving, and the related endothelial dysfunction and oxidative stress roles have been extensively investigated. However, most available data have been obtained before and after the dive, whilst, as far as we know, no data is available about what happens *during* the water immersion phase of dive. The scope of this study is to investigate the Nitrate and Nitrite (NO_X_) concentration and the total plasma antioxidant capacity (TAC) before, *during* and after a single SCUBA dive in healthy scuba diving volunteers, as well as to look for evidence of a possible relationship with venous gas bubble formation.

**Materials and Methods:** Plasma, obtained from blood of 15 expert SCUBA divers, 13 male and 2 female, was investigated for differences in NO_X_ and TAC values in different dive times. Differences in NO_X_ and TAC values in subjects previously known as “bubble resistant” (non-bubblers – NB) and “bubble prone” (Bubblers – B) were investigated.

**Results:** We found a statistically significant increase of NO_X_ plasma concentration in the “bottom blood draw” and in the “safety stop blood draw” as compared to the basal pre diving condition. We did not find any difference in NO_X_ plasma concentration between the basal value and the post diving samples. We did not find any significant statistical difference in TAC in the bottom blood sample, while the safety-stop and the post-dive samples showed higher TAC values compared with the basal value. We did not find any difference in NO_X_ and TAC mean values between non-bubblers and Bubblers.

**Discussion:** Our protocol, by including underwater blood drawing, allowed to monitor plasma NO_X_ changes occurred *during* diving activity, and not only by comparing pre and post diving values. It is particularly interesting to note that the increased NO_X_ values found at the bottom and at the safety stop were not observed at post dive sampling (T0, T30, T60), showing a very rapid return to the pre-dive values. In this preliminary study we did not find any relationship between bubble formation and changes in NO_X_ parameters and TAC response.

## Introduction

Nitric oxide (NO), defined in 1992 the “Molecule of the Year,” (Koshland, 1992) is known to play an important role in several physiological processes, particularly in the control of the cardiovascular system, and the blood flow and pressure (Lundberg et al., 2015).

However, the role of NO in human physiological and pathological responses is more complex, including its nature as a signaling molecule in several physiological mechanisms such as: central nervous system transmission and neuroprotection (Rand, 1992; Calabrese et al., 2007) immune system response (Bogdan, 2001), vascular remodeling (Witte and Barbul, 2002), and several other conditions (Yang et al., 2018).

Nitric oxide is largely released from the arterial endothelium that can be considered a real autocrine and paracrine organ, able to produce many other substances such as Prostacyclin, Endothelium Derived Relaxing Factor, Platelet Activating Factor, Angiotensin-Converting Enzyme and several other (Heitzer et al., 2001). As a consequence, NO is involved in many physiological processes such as: platelet activity, leukocyte adhesion, thrombosis, and in the development of atherosclerosis (Keaney and Vita, 1995; Tousoulis et al., 2010).

Based on these premises, it is easy to understand that endothelial dysfunction is involved in the majority of diseases affecting the vascular district, including the relationship with coronary artery disease and peripheral vasculature (Drexler, 1997; Pepine, 1998).

Recently more and more confirmations suggest a primary role of the increase of oxidative stress as the cause of endothelial dysfunction (Cai and Harrison, 2000).

Endothelial dysfunction and oxidative stress have been also investigated in diving (Brubakk et al., 2005; Obad et al., 2010) with particular regard to NO-related endothelial changes both in self-contained underwater breathing apparatus diving (SCUBA) (Theunissen et al., 2013a) and in Breath-hold diving (BH-diving) (Theunissen et al., 2013a,b).

Diving-related Venous Gas Emboli, frequently observed in divers, even when correct decompression procedures are followed (Giordano, 2005; Valic et al., 2005; Cialoni et al., 2017) could be a way to explain endothelial dysfunction after SCUBA diving (Nossum et al., 1999, 2002) as well as diving- related hyperoxia inducing an increase of reactive oxygen species (ROS) (Jamieson et al., 1986; Narkowicz et al., 1993).

Nitric oxide is difficult to quantify directly in blood due to its very short half-life (0.05–1.8 ms) (Liu et al., 1998). It’s preferable to measure stable NO derivative products (Tsikas, 2005) such as nitrates and nitrites (NO_X_), the final results of the NO oxidation pathway (Van Vliet et al., 1997). NO_X_ can support NO signaling under metabolic stress conditions through the activation of enzymatic and non-enzymatic reactions in tissues.

Nitric oxide is oxidized in the blood and tissues to form nitrate and Nitrite (Moncada and Higgs, 1993). The reaction of NO with oxyhaemoglobin produces nitrate and methaemoglobin (Moncada and Higgs, 1993), whereas the oxidation of nitrite occurs in the red blood cell and results in the formation of nitrate and methaemoglobin (Met-Hb) (Lundberg et al., 2008).

The oxidation of NO forms nitrite, a process that is catalyzed in plasma by the multi-copper oxidase and NO oxidase ceruloplasmin (Shiva et al., 2006). Nitrite is also formed in the body via the oxidation of NO or through the reduction of nitrate but this non-enzymatic reaction of NO with oxygen in tissues is relatively slow (Lundberg et al., 2008).

On the other hand, an indirect method to measure the ROS produced by cellular metabolism, environmental factors and the balance between oxidant and antioxidant agents is to investigate the total plasma antioxidant capacity (TAC) by the Ferric Reducing Antioxidant Power (FRAP) test (Benzie and Strain, 1996).

Although several experimental data converge in attributing to NO important roles in the physiology and pathophysiology of diving (Theunissen et al., 2013a) the supporting scientific evidence is still weak. However, most available data have been obtained on land (be it laboratory of field conditions) before and after the dive, but, as far as we know, no data is available on what happens during the water immersion phase of the dive.

The scope of this study is to investigate the NO_X_ concentration and the TAC before, *during* and after a single SCUBA dive in healthy scuba diving volunteers, as well as to look for evidence of a possible relationship with venous gas bubble formation.

## Materials and Methods

### Subjects and Diving Procedures

A total of 15 expert SCUBA divers, 13 male and 2 female, were studied during a single dive.

No subject reported previous episodes of decompression illness (DCI), historical or clinical evidence of arterial hypertension, cardiac, pulmonary or any other significant disease, none of them took prescription drugs, suffered any acute disease during the 15 days before the experiment, or reported assumption of anti-inflammatory drugs and exposure to high altitude in the 7 days before the experiment. All the divers received an explanation of the study’s purposes, risks and benefits, were familiarized with the experimental protocol and read and signed a specific informed consent form before the experiment.

Subjects were asked to avoid food rich in nitrate, such as red meat (Lundberg, 2009) and leafy green vegetables (Lundberg and Govoni, 2004) and moderate or intense exercise during the 48 h before the experiment.

The study was conducted in accordance with the Helsinki Declaration and was approved by the Ethical Committee of Università degli Studi di Milano, Italy (Aut. n° 37/17).

No Diver performed any compressed-gas diving or any Breath Hold Diving during 30 days before the day of the test.

The diving parameters (depth, diving time and Gradient Factor) were recorded for each dive using a diving computer with sampling rate set at 10 s. All the dive profiles were downloaded using the Subsurface software, exported in UDDF format and imported into the DAN Europe Diver Safety Guardian (DAN DSG) original and proprietary program to calculate the Maximum Gradient Factor reached during the dive. This Maximum value is generally reached at the end of the dive. The Gradient Factor measures the inert gas load in the diver’s tissues, according to the selected decompression algorithm. In our protocol, the maximum Gradient Factor, expressed as a percentage of the *M*-value, is computed using the Buhlmann ZH-L16 C model. This is a way to estimate inert gas supersaturation and to compare diving exposure in the different investigated subjects (Cialoni et al., 2017).

**FIGURE 1:**
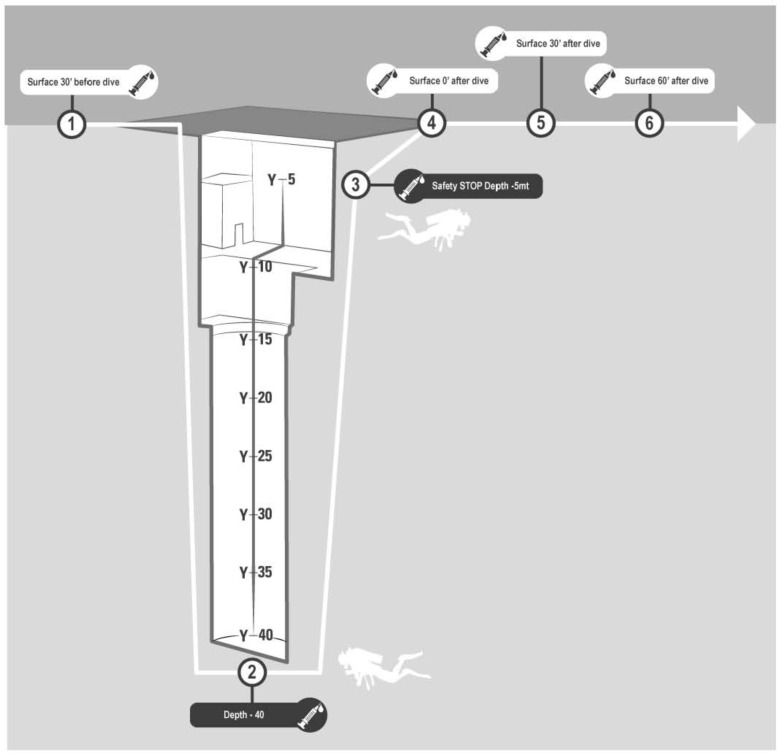
Venus blood samples were investigated in every subject: ✓30 min before diving (basal), ✓At −42 m pool bottom (diving 1), ✓During the safety stop at −5 m (diving 2), ✓Immediately at the end of diving (post 0), ✓30 min after diving (post 30), ✓60 min after diving (post 60).

### Materials and Protocol

A 2-way peripheral venous catheter was placed in the antecubital vein before the dive, wrapped in waterproof bandage and fixed with waterproof plaster. A 3-way stopcock was connected to the catheter and a IV. Cannula needle. A vacutainer tube with EDTA as anticoagulant was used to collect blood samples at each phase of the blood draw protocol. Blood samples were collected 30 min before diving (basal), at the pool’s bottom (−42 m) (diving 1), during the safety stop at −5 m (diving 2), immediately after the end of the dive (post 0), 30 min (post 30) and 60 min after surfacing (post 60) Figure 1.

The collected blood was immediately treated to obtain plasma; as far as the blood sample taken at bottom is concerned, it was put into a dry box and immediately taken from the bottom to the surface, by means of a rope hoisted in about 1 min by a dedicated person. The same procedure was applied for the blood collected at −5 m.

Plasma was separated from the cell component by centrifugation (3000 rpm for 15 min) and was refrigerated at 4°C. The plasma samples were then delivered to the Laboratory of Biochemistry of the Department of Health Sciences (DISS) of the Università degli Studi di Milano for analysis.

Plasma was investigated for:

✓ Differences in plasma concentration of Nitrate and Nitrite (NO_X_) at the above indicated different times of the blood sample collection.
✓ Difference of total antioxidant capacity in blood by FRAP assay.

Urine density, hemoglobin and haematocrit were also recorded and used to calculate changes in blood volume (BV), red cell volume (CV), and plasma volume (PV) before and after the dive series, using the Dill and Costill formula (Dill and Costill, 1974).

### Plasma NO_X_ Measurement

Before the analysis, 400 μl of sample were treated with 400 μl of acetonitrile (Romitelli et al., 2007) to precipitate proteins, and then centrifuged at 12,000 rpm for 10 min. NO_X_ were measured in the de-proteinised plasma. The method for detection of plasma NO_X_ was based on Griess’s reaction as index of NO concentration (Green et al., 1982). Standard solutions of NaNO_3_ in a concentration range from 5 to 200 μM were placed in a 96 wells polystyrene microplate to build the standard curve. The diluting medium was used as the standard blank. After loading the plate with standard solution or sample, addition of 100 μl 51 mM VCl_3_ to each well was followed by 25 μl of 87 mM sulfanilamide and 25 μl of 1 mg/ml N-(1-Naphthyl) ethylenediamine. After 50 min incubation at 55°C, the optical density was read at 540 nm in a Sunrise microplate reader (TECAN, Salzburg, Austria). Plasma NO_X_ levels were obtained by interpolation of standard nitrate curve.

### Plasma TAC Measurement

We also investigated the reducing ability of plasma by the FRAP assay, performed according to Benzie and Strain (1996) with slight modifications. Twenty microliters of plasma were added to 3 mL of a freshly prepared FRAP solution in glass test tubes in triplicate and the absorbance measured at 593 nm in a Uvikon 931 UV-VIS Spectrophotometer (Northstar Scientific, Bardsey, United Kingdom). After 5 min of incubation at 37°C against a blank of FRAP solution. Aqueous solutions of FeSO_4_ 7H_2_O (100–1000 μM) were used for the calibration and the TAC results were expressed as FRAP value [μM Fe (II)] of the Samples (Zarban et al., 2009).

### Echocardiography Protocol

Echocardiography was done before the dive and within 30 min maximum after the dive.

All echocardiographies were made using a commercially available instrument (MyLab 5, Esaote SPA, Florence, Italy) with a cardiac probe (2.5–3.5 MHz).

All echocardiograms were performed with the subject lying on the left side and breathing normally: recording time was 20 s, and all frames were saved in the hard drive for subsequent analysis.

Bubbles were graded according to the Eftedal and Brubakk (EB) scale, and with the consensus guideline of ultrasound use in diving research, as follows: (Eftedal and Brubakk, 1993; Møllerløkken et al., 2016).

0 – no bubbles;
1 – occasional bubbles;
2 – at least one bubble per 4 heart cycles;
3 – at least one bubble per cycle;
4 – continuous bubbling;
5 – “white out”; impossible to see individual bubbles.

After grading the divers, they were divided into two groups: subjects not showing bubbles or only solitary bubbles, non-bubblers (NB), and subjects showing consistent bubbles degree 2 or higher (B).

### Statistical Analysis

Data are presented as the mean ± standard deviation (SD) for parametric data and median and range for non-parametric data. Taking the pre diving value of NO_X_ and TAC as 100% the percentage of changes were calculated in each measurement foreseen by the protocol. They were analyzed by means of one sample *t*-test after the D’Agostino and Pearson normality test to assume a Gaussian distribution.

Differences between non-bubblers and Bubblers were investigated using the Mann–Whitney *U*-test for non-parametric data and two-sample (unpaired) *t*-test for parametric data both after Shapiro–Wilk normality test.

A probability lower than 5% was assumed as the threshold to reject the null hypothesis (*P* < 0.05).

## Results

A total of 15 experienced SCUBA divers, 13 male and 2 female, mean age 47.9 ± 10.7 years; mean height 176.7 ± 6 cm; mean weight 78.9 ± 13.2 kg, and BMI 25.18 ± 3.6 were studied (Table 1).

**Table 1:** We did not find any correlation between NOX changes and personal anthropometrical data.

***Anthropometric Data***		
Age_(years)_	47.9	±10.7
Height_(cm)_	176.7	±6
Weight_(Kg)_	78.9	±13.2
BMI_(Kg/m_^2^_)_	25.18	±3.6
***Characteristics of Dives***		
Diving profile_(meters)_	41.2	±0.6
Diving time_(minutes)_	42.5	±3.5
Gradient factor_(absolute)_	0.85	±0.02

**FIGURE 2:**
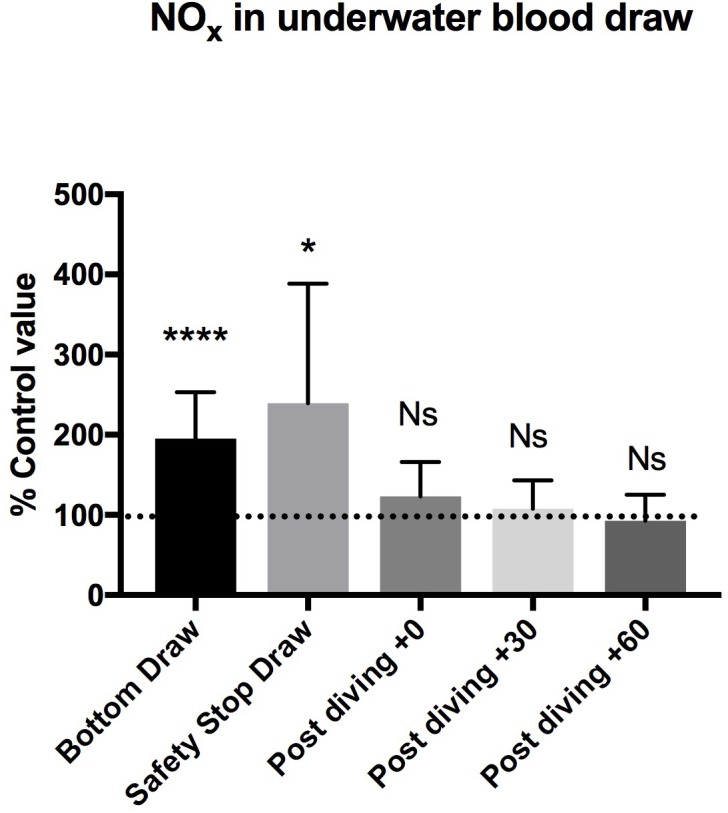
We found a statistically significant increase of NO_x_ plasma concentration in the “bottom draw” and during the “safety stop draw” compared to the basal condition. We did not find any difference in NO_x_ plasma concentration after diving. These changes were not confirmed immediately at the end of diving, showing a very rapid return to the pre-dive values, indicating the need for further real-time underwater investigation. ^∗^*P* < 0.05, ^∗∗^*P* < 0.01, ^∗∗∗^*P* < 0.001, and ^∗∗∗∗^*P* < 0.0001.

**Table 2:** NO_x_ and FRAP plasma concentration changes compared to basal values.

NO_X_ risk factor	Value	Ds/Range	% of control value	*P*-value
Pre diving 30 min	21.2 μM	(12.9–52.3)	100	Basal
Bottom draw	40.5 μM	(23.6–73.3)	195.2 ± 58%	*P* = 0.0001
Safety stop draw	43.6 μM	(24.0–87.4)	239.5 ± 149%	*P* = 0.01
T0 draw	24.9 μM	(10.8–55.2)	123.1 ± 43%	Ns
T30 draw	22.5 μM	(3.9–47.3)	107.6 ± 35%	Ns
T60 draw	22.1 μM	(6.7–55.0)	92.6 ± 32%	Ns
**FRAP risk factor**				
Pre diving 30 min	270.6 μM	±33.8	100	Basal
Bottom draw	272.2 μM	±92.6	103 ± 38.6%	Ns
Safety stop draw	337.9 μM	±123–1	127.1 ± 52%	Ns
T0 draw	296.0 μM	±39.0	109.4 ± 6.8%	*P* = 0.003
T30 draw	295.3 μM	±28.1	109.5 ± 5.0%	*P* = 0.0005
T60 draw	295.4 μM	±24.5	110 ± 10.3%	*P* = 0.02

The Diving profile showed a mean depth of 41.2 ± 0.6 meters a mean diving time of 42.5 ± 3.5 min with bottom time of 8.6 ± 1.9, ascent time 1.7 ± 0.3 m/s and a mean of GF of 0.86 ± 0.02 (Table 1).

Diving was performed in the swimming pool Y-40 (42 m depth), in thermal water at 34°C, and mean temperature, as recorded by diving computer, of 33.4 ± 0.7°C.

We found a statistically significant increases of NO_X_ plasma concentration in the bottom blood draw, +195.2 ± 58% of basal value (44.7 ± 14.2 μM) *P* = 0.0001, and in the safety stop blood draw +239.5 ± 149% of the basal value (50.25 ± 21.3 μM) *P* = 0.01, compared to the basal condition pre diving taken as 100% (24.6 ± 11.3 μM).

We did not find any difference in NO_X_ plasma concentration between the basal value and the post diving samples *P* > 0.05 (T0 = 29.1 ± 13.8 μM; T30 = 26.4 ± 13.3 μM; T60 = 23.6 ± 14.4 μM) (Figure 2 and Table 2).

NO_x_ values found before the diving exposure were in the normal plasma levels as compared to the no-diver population (20–40 μM) (Lundberg et al., 2008).

TAC was successfully investigated only in 9 of the 15 investigated subjects, on these we did not find any significant statistical difference in the bottom blood sample, while the safety-stop and the post-dive samples showed higher TAC values compared with the basal value (safety stop +127 ± 52.06%; T0 +109 ± 6.8%; T30 +109 ± 5.0%; T60 +110 ± 10.3%).

**FIGURE 3:**
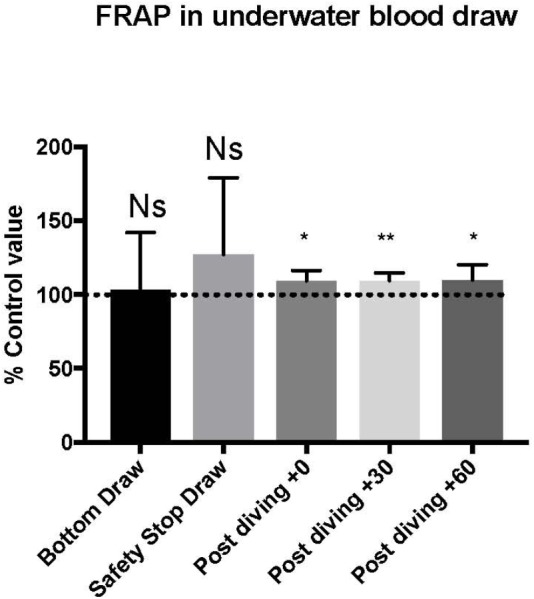
We found an increase in FRAP value compared to pre diving at the safety stop and at T0, T30, and T60. The safety stop increase is not statistically significant even if higher than the following values as follow: Safety stop = +127% of basal value; T0 = +109% of basal value; T30 = +109% of basal value; T60 = +110% of basal value.

Differences in T0, T30, and T60 were statistical significant (Figure 3 and Table 2). ^∗^*P* < 0.05, ^∗∗^*P* < 0.01, ^∗∗∗^*P* < 0.001, and ^∗∗∗∗^*P* < 0.0001.

We found four B (including two grade 2 and two grade 3 at EB Scale) vs. 11 NB.

We did not find any difference in NO_X_ and TAC mean between non-bubblers and Bubblers (NB vs. B) (Table 3).

Also we did not find any correlation between NO_X_ AGE and BMI.

Original data of NO_X_ plasma concentration are shown in Table 4.

We did not find any differences in hemoglobin (15.4 vs. 15.7), haematocrit (44.7 vs. 45.5); and urine density (1.015 vs. 1.014), before and after the dives series *P* < 0.05.

The BV the CV and the PV as per Dill and Costill formula was relatively unchanged (100 ml vs. 93.7 ml); (44.7 ml vs. 44.4 ml) (55.33 ml vs. 55.30 ml), respectively, the PV D was only 0.07%.

**Table 3:** No NO_x_ and FRAP differences were found between non-bubblers and bubblers.

NO_x_ risk factor	non-bubblers	Bubblers	*P*-value
Pre diving 30 min	24.4 ± 10.4 μM	25.0 ± 15.4 μM	Ns
Bottom draw	42.2 ± 13.3 μM	51.5 ± 16.5 μM	Ns
Safety stop draw	51.4 ± 21.7 μM	47.2 ± 23.1 μM	Ns
T0 draw	28.7 ± 12.7 μM	30.3 ± 18.7 μM	Ns
T30 draw	26.5 ± 14.6 μM	27.8 ± 12.7 μM	Ns
T60 draw	23.2 ± 14.0 μM	24.7 ± 17.7 μM	Ns
**FRAP risk factor**			
Pre diving 30 min	293.9 ± 45.9 μM	258.2 ± 17.5 μM	Ns
Bottom draw	299.0 ± 96.5 μM	261.5 ± 45.6 μM	Ns
Safety stop draw	243.4 ± 153.9 μM	359.1 ± 89.0 μM	Ns
T0 draw	290.2 ± 50.0 μM	291.8 ± 43.0 μM	Ns
T30 draw	291.2 ± 33.2 μM	285.4 ± 21.7 μM	Ns
T60 draw	298.6 ± 30.0 μM	288.3 ± 29.2 μM	Ns

**Table 4:** Original data of NO_x_ plasma concentration for each diver.

Diver	Pre diving	Bottom draw	Safety stop draw	T0 draw	T30 draw	T60 draw
1	21.03	51.46	30.94	30.94	26.8	31.3
2	25.52	59.87	26.64	26.64	45.89	32.29
*3*	*14.94*	48.13	24.05	24.35	19.48	14.22
*4*	*30.30*	51.97	67.39	54.79	47.29	36.24
5	52.33	68.13	43.24	43.53	45.01	55.01
6	15.52	45.52	66.64	19.68	17.29	16.94
7	21.96	38.86	87.37	19.07	19.81	23.48
8	47.72	73.29	70.66	55.24	46.16	46.33
9	12.92	23.61	74.27	24.92	3.93	6.68
10	16.21	33.29	63.19	10.77	18.86	6.86
11	24.61	29.27	33.47	11.91	16.08	12.28
12	19.95	40.50	43.59	19.72	19.72	11.57
13	24.43	33.01	27.77	36.19	22.51	22.15
14	19.86	36.85	66.72	22.72	23.30	12.64
15	21.23	36.21	27.85	36.06	24.41	26.51

## Discussion

Scuba diving exposes the human body to environmental stress conditions implying increased ambient pressure, pO2, physical efforts and breathing resistance (Doubt, 1996). Hyperoxia, due to the increased pO2, can lead to vasoconstriction and oxidative stress (Demchenko et al., 1985), which is at the base of endothelial dysfunction (Ferrer et al., 2007; Theunissen et al., 2013a,b).

Our protocol, by including underwater blood drawing, allowed to monitor plasma NO_X_ changes occurred *during* diving activity, and not only by comparing pre and post diving values.

It is particularly interesting to note that the increased NO_X_ values found at the bottom and at the safety stop were not observed at post dive sampling (T0, T30, T60), showing a very rapid return to the pre-dive values. These changes could not have been identified by the usual pre and post dive tests, suggesting the need to further develop underwater real-time monitoring protocols.

As already shown by other investigations of physiological changes in extreme environmental conditions (Cialoni et al., 2014, 2015), there are often potentially important differences between real conditions and simulated studies or models that not always permit a correct interpretation of the “temporal gap” between “pre” and “post” measurements. Studies like this one, that directly investigated this “black box time gap,” represent the challenge of future studies in scuba diving.

Our protocol investigated NO_X_ changes to evaluate the NO related vascular response and TAC was used to evaluate the total antioxidant activity of plasma.

We showed important NO related changes during diving that return to normality immediately after diving. This data on the one hand confirm an important vascular response during diving in which NO appears to play a primary role as a vascular mediator, and indirectly confirm a possible diving related endothelial dysfunction (Theunissen et al., 2013a). On the other hand, we showed a rapid return to the NO pre diving values immediately at the end of diving exposure, confirming these physiological changes during diving as probably related to the increase of ambient pressure given their rapid return to normal values when the diving exposure ended.

The limited changes in the plasma volume found after diving compare to the values recorded before the diving, maybe linked with the particular environmental condition of test (without any cold stimulus), suggest a marginal rule of the hemoconcentration in the NO_x_ and TAC values found in our test.

Parallelly we found a significant increase of TAC values only in the final and post diving phases.

Even if this part of our tests needs further investigation and the result must be considered preliminary, data seem to confirm that the dive causes an oxidative stress (Jamieson et al., 1986; Theunissen et al., 2013b) possibly activating a complex endogenous antioxidant system including enzymes such as Superoxide dismutase (SOD), glutathione peroxidases and catalase; proteins such as albumin, ceruloplasmin and ferritin (Cao and Prior, 1998), and an array of small molecules including ascorbic acid, uric acid (Ames et al., 1981), bilirubin (Stocker et al., 1987; Belanger et al., 1997), α-tocopherol, β-carotene, ubiquinol-10, reduced glutathione (GSH), methionine (Cao and Prior, 1998). This activation can explain the increased antioxidant capacity that we found at the end of the dive and after diving.

Scuba diving can activate the antioxidant defenses to control vascular oxidative stress due to increased O_2_ level associated to hyperbaric conditions and change the vascular endothelial growth factor metabolism, with the activation of a signaling cascade resulting in a stimulation of tissue resistance to diving-derived oxidative stress (Sureda et al., 2012).

In this preliminary study we did not find any relationship between bubble formation and changes in NO_X_ parameters and TAC response. If these data will be confirmed the role of oxidative stress and endothelial dysfunction may not be of particular importance in the pathogenesis of bubble formation, as a link between bubbles and DCI, and the role of these factors in the development of DCI could not be decisive.

This study represents a pilot test and has some important limitations for which further investigation is needed to confirm the preliminary results.

The first limitation is related to the particular context where the tests were done, the Y-40 swimming pool represents in fact a very particular dive-site, especially for the warm water temperature allowing to dive without a diving suit that, even if probably not causing real changes in the body temperature of diver (for the warm water temperature) and being exceptionally helpful as a test environment, is anyway different as compared with real diving conditions (sea or fresh water).

We should also point out that NO_X_ changes could be partly due to the known pro-inflammatory effect of diving and the observed increase of circulating MPs post diving; these aspects deserve further investigation and are part of our continuing field research action.(Thom et al., 1985).

This aspect requires to repeat the protocol in real diving condition to confirm the observed changes.

The low numbers of investigated subjects is a further limit.

But without any doubt the most important aspect that emerges from our study is the incredible celerity with which the value of NO_X_ returns to the normal range after the dives.

In any ordinary test, performed before and after diving, we could not have seen any such change and we would have concluded that the investigated substances don’t change during diving, while this protocol clearly shows the existence of very rapid changes during the underwater phase of the dive.

## Conclusion

We showed a statistically significant increases of NO_X_ during a single SCUBA dive, indirectly confirming an important diving related vascular involvement in 15 voluntary divers. The NO_X_ value return to basal conditions immediately after diving shows a very rapid control mechanism.

We also found an increase of the TAC after diving, confirming an oxidative stress during diving.

Both NO and TAC changes do not seem to have any relationship with inert gas bubble formation.

Further investigations are necessary (and planned) to confirm our preliminary results.

## Ethics Approval and Consent to Participate

All experimental procedures were conducted in accordance with the Declaration of Helsinki (World Medical Association) and were approved by the Ethical Committee of Università degli Studi di Milano, Italy (Aut. n° 37/17). All methods and potential risks were explained in detail to the participants. All personal data were handled.

## Author Contributions

All authors were involved in the conception and design of this work and contributed to the process of writing and approval of the final manuscript. DC implemented the systematic search strategy, extracted and analyzed the data, and wrote the first draft. AB was involved in the conception and design of this work, reviewed the critical appraisal of selected articles, and assisted with the compilation of the systematic review. MP extracted and analyzed the data and reviewed the manuscript. The entire process was supervised by MS and AM.

## Conflict of Interest Statement

The authors declare that the research was conducted in the absence of any commercial or financial relationships that could be construed as a potential conflict of interest.
